# Laboratory-based X-ray spectrometer for actinide science

**DOI:** 10.1107/S1600577523006926

**Published:** 2023-09-22

**Authors:** Daniil Novichkov, Alexander Trigub, Evgeny Gerber, Iurii Nevolin, Anna Romanchuk, Petr Matveev, Stepan Kalmykov

**Affiliations:** a Department of Chemistry, Lomonosov Moscow State University, Leninskie Gory 1-3, Moscow 119991, Russian Federation; b National Research Centre Kurchatov Institute, Ploshchad Akademika Kurchatova 1, Moscow 123182, Russian Federation; Bhabha Atomic Research Centre, India

**Keywords:** laboratory-based, X-ray spectrometers, X-ray absorption spectroscopy, X-ray emission spectroscopy

## Abstract

X-ray absorption and emission spectroscopies nowadays are advanced characterization methods for fundamental and applied actinide research. One of the advantages of these methods is to reveal slight changes in the structural and electronic properties of radionuclides. The experiments are generally carried out at synchrotrons. However, considerable progress has been made to construct laboratory-based X-ray spectrometers for X-ray absorption and emission spectroscopies. Laboratory spectrometers are reliable, effective and accessible alternatives to synchrotrons, especially for actinide research, which allow dispensing with high costs of the radioactive sample transport and synchrotron time. Moreover, data from laboratory spectrometers, obtained within a reasonable time, are comparable with synchrotron results. Thereby, laboratory spectrometers can complement synchrotrons or can be used for preliminary experiments to find perspective samples for synchrotron experiments with better resolution. Here, the construction and implementation of an X-ray spectrometer (LomonosovXAS) in Johann-geometry at a radiochemistry laboratory is reported. Examples are given of the application of Lomonosov­XAS to actinide systems relevant to the chemistry of *f*-elements, the physical chemistry of nuclear power engineering and the long-term disposal of spent nuclear fuel.

## Abstract 2

Experiments demonstrate that laboratory X-ray sources possess adequate power for conducting both X-ray absorption spectroscopy and X-ray emission spectroscopy measurements in the examination of actinides. Furthermore, the obtained spectra from these measurements are in agreement with synchrotron data.

## 1. Introduction

X-ray absorption spectroscopy (XAS) and X-ray emission spectroscopy (XES) are effective and non-destructive methods of investigating chemical state, in which samples may be in the gas phase, solutions or solids (Triguero *et al.*, 1998 ▸; Bianconi, 1980 ▸). Generally, X-rays from synchrotron radiation sources are used for XAS experiments. There are many synchrotrons worldwide, varying in beam energy, flux and brilliance; however, most of them do not support experiments with highly radioactive samples (Willmott, 2011 ▸). With new technological solutions arising in the field of X-ray sources (X-ray tubes), optics and detectors, the use of effective and compact laboratory X-ray spectrometers has become possible, making routine XAS and XES measurements available for scientists. From the instrumental point of view, a laboratory spectrometer consists of a crystal monochromator, a detector and an X-ray tube. These elements can be assembled in different geometries, the most common being Johansson (Johansson, 1933 ▸), Johann (Johann, 1931 ▸) and von Hamos (von Hámos, 1932 ▸).

In recent years, laboratory X-ray spectrometers have become commercially available; for instance, the commercial spectrometer QuantumLeap-H2000 from Sigray (https://sigray.com/quantumleap-h2000/), equipped with a high brightness source, operating in the spectral range from 4.5 keV to 25 keV. Johansson geometry is realized with an angle range from 55° to 85°, approaching the backscatter angle. Another example of commercial spectrometers is easyXAFS300+ from easyXAFS (Seidler *et al.*, 2014 ▸), well suited for XAS and XES experiments. It is realized in Johann geometry with an energy range from 5 to 18 keV and has a 1.2 kW X-ray tube with liquid cooling. Non-commercial spectrometers with different types of geometries are also known. For example, Błachucki *et al.* (2019 ▸) reported a spectrometer for simultaneous XAS and XES experiments with application of a double von Hamos crystal monochromator; the X-ray tube XOS X-beam Superflux is used as an X-ray source with a power rating of 50 W. XAS/XES laboratory spectrometers are continually being upgraded and improved. Although the spectrometer is essentially unchanged technically, Jahrman *et al.* (2019 ▸) reported great performance improvements by reduced degrees of freedom of the motorized platforms, increased flux and a wider Bragg angle, which allows the extended X-ray absorption fine-structure region to be measured over a larger energy range. One of the biggest centers for X-ray spectroscopy, located at the X-ray laboratory at the Department of Physics of the University of Helsinki (https://www.helsinki.fi/en/researchgroups/x-ray-laboratory), is working on the development of a wide range of X-ray spectrometers. For example, the HelXAS spectrometer was developed in 2017, and allows studying the 3*d* metals from Cr to Zn (Lahtinen *et al.*, 2022 ▸) and 4*f*-elements, such as Ce and Nd, and allows the local environment of actinides to be investigated, such as U or heavy metals such as Mo and Zr (Honkanen *et al.*, 2019 ▸). In addition to X-ray absorption spectroscopy, the X-ray spectroscopy center has recently implemented near-edge structure-based monochromatic computed tomography using a laboratory X-ray spectrometer (Honkanen & Huotari, 2023 ▸). Zimmermann *et al.* (2020 ▸) summarized and commented on the most notable developments in experimental laboratory setups – von Hamos and Johann/Johansson type setups. Since then, there have been notable changes in recent years with respect to laboratory X-ray spectroscopy setups.

Practically, X-ray spectroscopy is used in many fields of science, including chemistry (Szlachetko & Sá, 2016 ▸; Tromp *et al.*, 2002 ▸; Tromp, 2015 ▸), physics (Rovezzi & Glatzel, 2014 ▸; de Groot & Kotani, 2008 ▸) and materials science (Boscherini, 2015 ▸). Bes and co-workers were the first to report XAS measurements on radioactive U systems at the U *L*_3_-edge (Bès *et al.*, 2018 ▸) collected by laboratory X-ray spectrometer (Bès *et al.*, 2018 ▸). This example showed that laboratory-based spectrometers have a great capacity in the investigation of actinide materials (Kvashnina & Butorin, 2022 ▸; Kvashnina & de Groot, 2014 ▸; Kvashnina *et al.*, 2013 ▸, 2014 ▸, 2019 ▸). One of the advantages of using laboratory X-ray spectrometers in the actinide field is that the time allotted for the experiment is not limited, as it is at a synchrotron source, that restricts many scientific investigations that need to be done in a short time.

Synchrotrons enable the study of uranium and thorium and are widely used in scientific research (Caciuffo *et al.*, 2023 ▸; Kvashnina & Butorin, 2022 ▸; Kvashnina *et al.*, 2014 ▸, 2022 ▸; Caciuffo & Lander, 2021 ▸; Krot *et al.*, 2022 ▸; Gutorova *et al.*, 2022 ▸). However, while studying compounds of transuranium elements such as neptunium, plutonium, americium and other elements, special safety measures must be taken and they can be studied in a sufficient quantity only on specialized synchrotron beamlines. It should be noted that obtaining synchrotron radiation time is difficult. (Sitaud *et al.*, 2012 ▸; LLorens *et al.*, 2014 ▸; Borca *et al.*, 2009 ▸; Rothe *et al.*, 2012 ▸; Zimina *et al.*, 2016 ▸, 2017 ▸; Scheinost *et al.*, 2021 ▸). Furthermore, special safety precautions required for the radioactive samples result in high expenses, additional transport and experimental limitations. Transport complicates experiments with short-lived radionuclide compounds, as the procedure of sample delivery from the laboratory to the synchrotron is generally time-consuming. Moreover, it takes effort to obtain a relevant signal even in *ex situ* experiments for low concentrated samples at synchrotrons, while *in situ* measurements, demanding additional technical setup, are extremely challenging.

Here we present an X-ray spectrometer, named LomonosovXAS, which is dedicated to actinide science. It has been developed, produced and tested in the X-ray Laboratory at the Department of Physics of University of Helsinki and has been installed at the Radiochemistry Division of the Chemistry Department of Lomonosov Moscow State University (LMSU). We show here an X-ray spectrometer that is realized in Johann geometry with one spherically bent crystal-monochromator and detector, and that is able to record XAS and XES data on radioactive materials.

## 2. Experimental details

### 2.1. Spectrometer description

**Figure 1:**
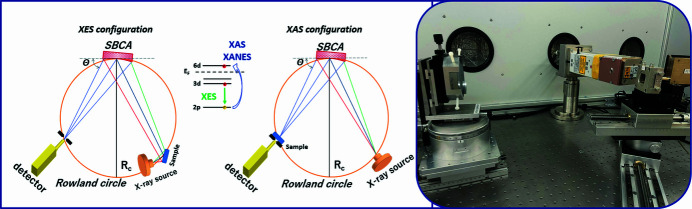
Schematic representation of the laboratory X-ray spectrometer for XES and XAS modes, as well as a photograph of the spectrometer in the XAS configuration. In the absorption mode the sample can be both on the detector and on the X-ray tube, while in XES mode the position of the sample remains fixed. SBCA: spherically bent crystal analyser.

Figure 1 ▸ shows schematic diagrams of an X-ray laboratory spectrometer in two configurations, XAS and XES, as well as an illustration of the absorption processes when the spectrometer is operated in the XAS and XES modes. XAS is based on the absorption of X-rays by an element, which leads to the knocking out of an electron to unfilled levels and the formation of a vacancy. XES observes the decay of the previously created central hole through the process of radiative decay from the occupied upper shell, so XES examines the occupied levels. XES is a complementary technique to XAS, and this allows us to obtain information about occupied and unoccupied states and the entire electronic structure of the material under investigation with one device.

The three main components of a spectrometer are X-ray source, spherically bent crystal-monochromator and X-ray detector located on the Rowland (Johann, 1931 ▸) circle with 0.5 m or 1 m radius (*R*_c_). A glass diffraction X-ray tube (XRD Eigenmann GmbH) with a relatively sharp focus, high intensity and good resolution of the diffraction lines is used as the X-ray source. The X-ray tube is equipped with a Be window and with a focus spot of 6° at the incident angle and provides a point focus of 0.4 mm × 0.8 mm. The power of the tube is 1.5 kW, and the anode material is chosen to be silver due to the lack of characteristic X-rays in the energy range 3–22 keV. Beryllium foil, of thickness 250 µm, is used as the exit window, suppressing radiation below 4 keV, and is therefore perfectly suited for the Bremsstrahlung energy range of 4–20 keV. A spherically bent crystal [of diameter 100 mm and bending radius 0.5 or 1.0 m (though most of the time the 0.5 m option is used)] reflects and monochomatizes X-rays from the X-ray tube to the detector, thus the optical system makes it possible to install the sample both at the exit of the X-ray tube and at the entrance to the detector. Analyzers consist of a thin strip of Si or Ge cut into 15 mm-width strips and stuck onto a curved holder pad of radius 2*R*_c_. To select the angle with respect to the incident beam, the crystal reflects the corresponding spectral component in accordance with Bragg’s law. A silicon drift detector (Amptek Inc.) with integrated electronic signal processing is used to record the XAS and XES spectra. Such detectors are commonly used today in X-ray spectrometers as counters of the analyzed X-ray signal. The entire detector setup is packed into an aluminium box (7 cm × 10 cm × 2.5 cm) with a chip thickness of 500 µm (70 mm^2^ active area). The 150 eV energy resolution makes it easy to reject any harmonics and most of the background fluorescence for high-precision measurements.

The spectrometer can be used in XAS and XES configurations. The only difference is that for XES mode the sample must be placed in a Rowland circle in the X-ray beam at 45° and we must excite above the absorption edge of the element under study; this is worth remembering when setting the X-ray tube parameters. For XAS, the sample is located at the exit of the X-ray tube or on the detector, but care is required to ensure that the beam is correctly aligned with the sample while the X-ray tube parameters are set to the absorbed energy of the element under study.

The basic idea in both measurements remains unchanged – the crystal-monochromator selects the energy, absorption energy or emission energy, and then part of the beam is reflected and focused in accordance with Bragg’s law (Bragg & Bragg, 1913 ▸),

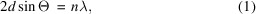

where Θ is the Bragg angle, *d* is the interplanar distance, *n* is a positive integer and λ is the wavelength of the incident wave. The interplanar distance can be calculated from the Miller indices of the crystallographic planes (*h*, *k*, *l*) and the lattice parameter *a*,

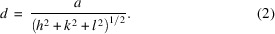

This formula is applied to the crystals which are commonly used as monochromators or analyzers to select or analyze X-rays of specific wavelengths. They can generate monochromatic X-ray beams reflecting or transmitting X-rays at specific Bragg angles. However, the energy-dispersive crystals can also be used in X-ray spectrometers to separate and analyze X-rays based on their energy or wavelength. These crystals disperse X-rays over a range of energies rather than selecting a specific wavelength. Overall, cubic crystals are used for X-ray monochromatization or analysis of specific wavelengths, while dispersive crystals are employed for energy-dispersive analysis of X-ray spectra.

The tabulated values of *d* for Si and Ge crystals are 5.4309 Å and 5.6574 Å, respectively.

The correspondence between wavelength λ (Å) and photon energy *E* (keV) can be obtained from the equation 

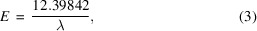

where 12.3984 is the conversion 1 keV = 12.984 Å. In that case Bragg’s law can be written as

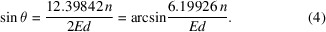

In order to introduce XES, we choose the following modification of the optical scheme: the sample is placed just after the X-ray source so that the angle between the incident X-ray beam and emitted radiation is 45°. The positions of the sample, crystal-monochromator and detector remain on the Rowland circle with radius *R*_c_. A crystal with a fixed radius of 2*R* reflects fluorescence from the sample to the detector. During the XES measurements, it is necessary to change the angle of the crystal-monochromator together with all other distances to follow the Rowland geometry (*cf.* Fig. 1 ▸). The position of the sample during the measurement does not change, and the distance between the sample and the crystal remains equal to the distance from the crystal to the detector during the scan.

While planning an experiment, one needs to select the crystal-monochromator to have the highest Bragg angle possible for the best energy resolution. For example, Np can be measured with Ge [1600] with Θ = 84.62°, or Ge [999] with Θ = 75.93°, which is less favorable. At an angle of 75°, the resolution will be 2.66 times lower [cot(75.93)/cot(84.62) ≃ 2.66]. Unfortunately, bent crystal-monochromators have aberrations. Experiments in Johann geometry decrease resolution at low Bragg angles compared with Johansson geometry. The main type of aberration in this case is astigmatism. The beam, reflected from the crystal, becomes more elongated and curved, resulting in decreased signal and resolution (Bergmann & Cramer, 1998 ▸). At higher Bragg angles, the effect of aberration is minimal; therefore, the total energy resolution is better. The intrinsic resolution of the analyzer Δ*E*_a_ is determined by the incident energy *E*_i_, the angular (Darwinian) reflection width *W* of the crystal reflection and the Bragg angle Θ,

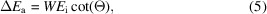

where Θ is the Bragg angle (incident) and *E*_i_ is the energy of the incident photons. For Ge[1600], *W* = 4.28 µrad, and for Si[999], *W* = 1.29 µrad (Gog *et al.*, 2019 ▸). It can be seen that the energy resolution of such an analyzer is best for reflections with small Darwinian widths and conditions close to backscattering, where the Bragg angle is close to 90°, and the cotangent consequently tends to zero (Gog *et al.*, 2013 ▸).

In order to optimize X-ray spectrometers both at synchrotrons and in the laboratory, it is crucial to understand the relationship between the diffraction properties and the mechanical deformation of spherically curved analyzer crystals. Generally, for 0.5 m analyzers, bending stresses can significantly reduce the resolution. However, this can be minimized by using strip-bent or diced analyzers. Rovezzi *et al.* (2017 ▸) discussed the performance of various analyzer crystals, comparing bent crystal and strip-bent crystal analyzers, as well as different radii of curvature for both 0.5 m and 1 m crystals. Their findings revealed that 0.5 m analyzers collect more photons and have energy resolutions close to the limit, considering both their own and geometric contributions. The increase in intensity compared with a 1 m-long bent-stripe crystal ranges from 2.5 to 4.5. Furthermore, Honkanen and Huotari (Huotari *et al.*, 2005 ▸, 2006 ▸; ; Honkanen & Huotari, 2021 ▸) explored diced analyzers and provided detailed models for predicting the intensities of diffracted X-rays. Their study was supported by comparison with experimental data, confirming the efficacy and performance of diced analyzers in practice.

**Figure 2:**
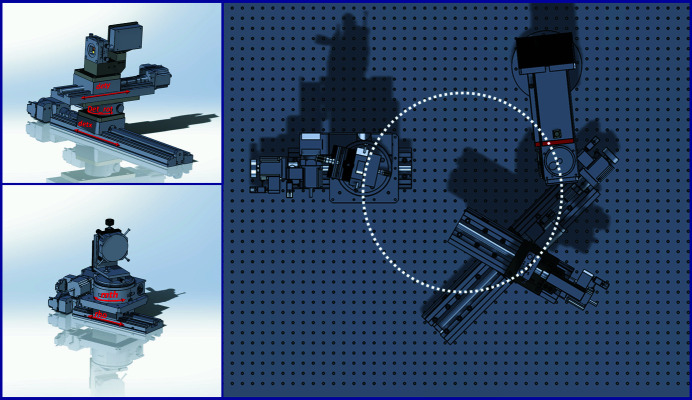
CAD model of the X-ray spectrometer with X-ray tube, crystal-monochromator and detector.

Figure 2 ▸. shows a CAD model of the laboratory X-ray spectrometer. In this experimental setup, the position of the X-ray source is fixed. The monochromator and detector move during the energy scan to maintain Rowland circle geometry. All spectrometer parts (detector and monochromator) are mounted on motorized stages, for which five motors are used (fabricated by xHuber Diffraktionstechnik, https://www.xhuber.com/en/). If Bragg’s angle of the monochromator (named ‘mth’) changes, the distance to the source (named ‘rho’) varies as well and the focus spot is moved in space. The detector should be moved to trace the focus X-ray spot at all Bragg angles. Two motors (named ‘detx’ and ‘dety’) are responsible for the detector movements in two perpendicular directions. The motor ‘detrot’ is responsible for the detector rotation to point at the center of the monochromator and its value is always equal to 2θ. The control computer is operated under OS Linux, using the Certified Scientific Software *spec* (https://www.certif.com/). The main parameters of the laboratory X-ray spectrometer are listed in Table 1 ▸.

To make correct XAS spectrum measurements with the spectrometer, four scans of the monochromator are required: two with a sample, with detector focused (*I*) and detector background measurement (*I*_bkg_); and two without a sample (*I*_0_ and *I*_0,bkg_). The background measurement is performed by offsetting the detector, typically by 10 mm, and measuring the same energy range as for *I* and *I*_0_. The background spectra *I*_bgk_ and *I*_0,bgk_ usually do not have any structure. Then the absorption coefficient can be found as

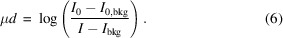

Due to the nonlinear dependence of μ_*x*_ on beam density, it becomes necessary to determine and subtract background levels from the actual signals. Background levels are caused by scattering in the air and components in the fluorescence spectrometer that create elastic and inelastic scattering. In addition, different sample holder designs result in different background values at the detector. To obtain an accurate estimate of the background at the focal spot, the detector offset is measured on one side and then on the other side of the beam focus. The average value of the background signals is approximated by a low-order polynomial, after which the resulting background (denoted as bkg) is subtracted from the recorded signals *I* and *I*_0_.

Recently, Bes and co-authors (Bes *et al.*, 2021 ▸) suggested an alternative approach for simultaneous *I*_0_ and *I* measurements based on the use of the harmonics, naturally occurring when crystal-analyzers are used. Two independent measurements instead of one increase the total time for the data acquisition as well as the risk that *I*_0_ instability will cause glitches. Besides that, if the sample environment is complex [such as an operand spectro-electrochemical cell (Kapaev *et al.*, 2022 ▸) or other instruments, such as actinide *in situ* cells], the removal of the sample and its environment may have an unknown effect on the spectrum. The reliability of this approach was confirmed by Co *K*-edge experiments, conducted with metal Co foil. It was found that application of higher or lower harmonics reflected by the crystal-monochromator is a good equivalent of *I*_0_ measurements. It is required to differ the harmonics to separate their contributions. This can be done, for example, with an energy-dispersive detector.

**Figure 3:**
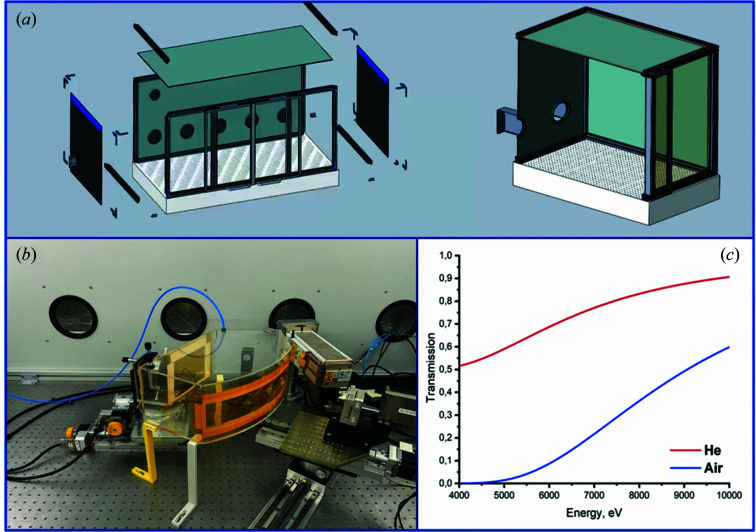
(*a*) CAD model of the security module, in which the left-hand part of the figure shows the separated parts in the assembly, and the right-hand part is a sectional view of the introductory labyrinth type channel. (*b*) Helium chamber. (*c*) Simulations of X-ray beam transmission through the air and helium.

The LomonosovXAS spectrometer has a custom-made shielding unit to protect personnel from radiation [*cf.* Fig. 3 ▸(*a*)]. The design ensures continuity of protection against radiation over the entire outer surface of the shielding unit. The shielding module is equipped with a gliding X-ray protective door with X-ray protective plexiglass (thickness in lead equivalent 0.5 mm) supplied with electromechanical locks, limit switches, a control unit, lamps and ventilation with mechanical awakening. The back side of the shielding unit is equipped with five labyrinth-type channels for input and connection of various electronics, gas and water tubes (right side of Fig. 3 ▸).

The spectrometer is equipped with a helium chamber [Fig. 3 ▸(*b*)], which is used for the measurements in the region 4–10 keV and helps to reduce air absorption. The helium chamber contains several windows: the cutouts on the front side are oriented at the height of the source and the detector, and the rectangular cutout on the far side allows X-rays to reach the crystal-monochromator. Each window is covered with a polyimide film (Kapton) attached to the frame of the helium box. Figure 3 ▸(*c*) shows a comparison of simulations of the X-ray beam passing through air and through helium. The simulation assumes that the optical path of the X-ray beam is 1 m, given the distances from the X-ray tube to the monochromator crystal (0.5 m) and from the monochromator crystal to the detector (0.5 m). However, the helium box does not cover the entire length of the optical path of the X-ray beam (0.3 m), which leads to loss of intensity. Consequently, the calculations were performed for the actual length of the X-ray beam path through the helium box. To increase the intensity and reduce the losses there is the possibility of using inflatable helium bags, which will cover almost the entire volume of the optical circuit. Data were simulated using the Center for X-ray Optics web program *CXRO* (Henke *et al.*, 1993 ▸) with which the user can easily enter variables for the simulation of the X-ray transmission efficiency of solids (*e.g.* chemical formula, density and thickness) and gases (*e.g.* chemical formula, pressure, temperature and path length). The data show that the use of helium (instead of air) improves the efficiency of X-ray transmission in the energy range from 4 to 10 keV.

### 2.2. Sample preparation

Sample preparation for XAS measurements is quite straightforward; however, there are several issues that need to be taken into account. It is extremely important to make a homogeneous sample and position it at the height of the beam. Moreover, the sample size should match the beam width. Sample concentration prescribes the best measurement mode. In general, dilute or thick samples are measured in fluorescence mode, while concentrated samples are measured in transmission mode. The sample thickness for transmission measurements can be estimated in advance by special software, for instance, *Hephaestus* from the *IFEFFIT* package (Ravel & Newville, 2005 ▸). General practice is to estimate the absorption length value based on the density and chemical formula of the studied material. It is better not to exceed values of 1 to ∼1.5 times the absorption length. If the sample is too thick, the transmitted intensity will be too low to collect a proper signal (Calvin, 2013 ▸). The XES mode does not require complex sample preparation. In this measurement mode the thickness of the sample does not affect the quality of the measurement, since there are no self-absorption effects. It is important to note that self-absorption is a phenomenon that can occur when the emitted radiation is re-absorbed by the emitting material, resulting in a decrease in the observed radiation intensity. It is known that self-absorption has no significant effect on the radiation band under study, which means that the emitted radiation can leave the material without significant reabsorption (de Groot & Kotani, 2008*a* ▸).

In this subsection, we briefly describe the preparation of a radioactive sample. A uranium dioxide sample, obtained from the UO_2_ reference, is made by pressing industrially obtained uranium dioxide powder into a 6 mm-diameter tablet. To prepare the tablet, UO_2_ particles 0.2–1.6 mm in size were ground in a mortar and mixed with cellulose. The industrial uranium dioxide was obtained from UF_6_ using the gas-flame method, followed by annealing under reducing conditions at 600–650°C. Synthesis of the ThO_2_ sample was carried out as follows: in a first step, a white precipitate of nanocrystalline ThO_2_ was obtained by chemical precipitation from 1 *M* aqueous thorium (IV) nitrate solution [Th(NO_3_)_4_·5H_2_O] and 3 *M* ammonia solution. The resulting precipitate was separated from the mother liquor, washed repeatedly with MilliQ water by centrifugation, and dried at 40°C for 24 h. Next, the dried ThO_2_ powder was annealed in a muffle furnace at 1000°C for 12 h. The heating rate was 10°C min^−1^. Around 2 mg of ThO_2_ powder was mixed with cellulose and pressed into a thin pellet, that was then placed in a washer and glued with a thin layer of Kapton (of 25 µm). The plutonium stock solution contained two isotopes, ^239^Pu (99.99 mass%) and ^238^Pu. The PuO_2_^2+^ solution was prepared by fuming a stock solution with concentrated perchloric acid for several hours. For obtaining a solution of NpO_2_^+^, a small aliquot of NaNO_2_ was added to the stock which contained only ^237^Np. The oxidation state of both actinides was proved by UV–Vis spectroscopy (Shimadzu UV-1900i). The radioactivity of each sample is shown in Table 2 ▸.

## 3. Results and discussion

### 3.1. XAS configuration

The difference in the behavior of photoelectrons with different energies during scattering is the reason that the absorption spectra have to be divided into two parts. The first is a low-energy region called XANES (X-ray absorption near-edge structure), which corresponds to photoelectron energies up to ∼30 eV (and in some cases up to 50 eV). The second is a high-energy region called extended X-ray absorption fine structure (EXAFS), where the main contribution to absorption comes from a single (or sometimes multiple) scattering of a photoelectron.

**Figure 4:**
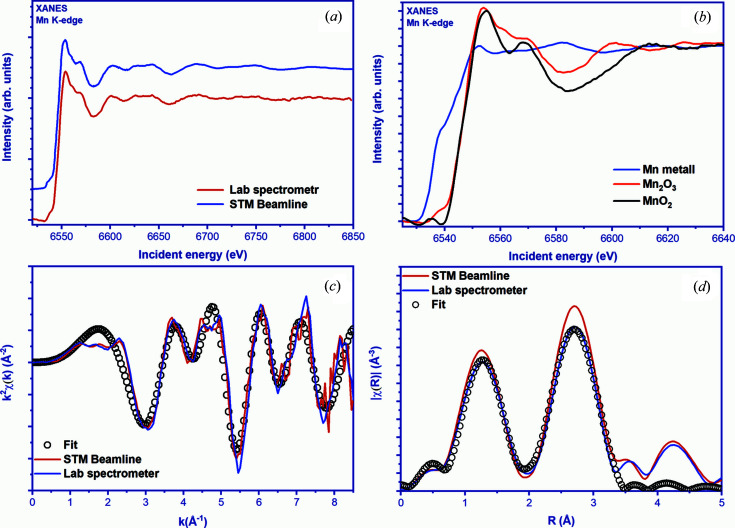
(*a*) Comparison of the Mn *K*-edge XANES spectra of Mn_2_O_3_ collected by the laboratory spectrometer (red) and synchrotron radiation (blue, measured on the STM beamline of the Kurchatov Scientific Center). (*b*) *K*-edge XANES on manganese oxides. (*c*) Oscillating parts and Fourier transforms of X-ray spectra of Mn_2_O_3_. (*d*) Fourier transforms of X-ray spectra of Mn_2_O_3_.

The XANES spectroscopy part includes different regions. (i) A sharp rise in the experimental X-ray absorption spectrum, called an edge. This occurs because, at energies below the edge, X-ray photons do not have enough energy to excite electrons from any particular orbital, but above it they do [in the case of Mn it is a transition from the 1*s* core state to the 4*p* conduction band (de Groot & Kotani, 2008 ▸) at the energy 6539 eV (Bearden & Burr, 1967 ▸)]. (ii) The pre-edge region for 3*d*-transition metals corresponds to the advancement of an electron from the 1*s*-orbital of the *K*-shell to the 3*d*-orbital. It refers to the pure quadrupole or a mixture of quadrupole and dipole transitions (Glatzel & Bergmann, 2005 ▸; de Groot & Kotani, 2008 ▸; de Groot *et al.*, 2005 ▸; Cabaret *et al.*, 2010 ▸). Pre-edge, main edge and post-edge regions can be analyzed by electronic structure calculations using different user-friendly codes (*FEFF*, *FDMNES*, *ORCA*, *Wien2K*, *OCEAN*, *QUANTY*, *etc*.) or codes developed in-house. Analysis of the XANES spectra makes it possible to obtain information on the oxidation state and local geometry near the absorbing atom (de Groot *et al.*, 2009 ▸). The position of the absorption edge on the absolute energy scale is very sensitive to the oxidation state [*c.f.* Fig. 4 ▸(*b*)] of the absorbing atom (Mansour *et al.*, 1994 ▸). The XANES spectrum is collected in a relatively narrow energy range, which reduces the measurement time, compared with what is required for EXAFS measurements.

EXAFS spectroscopy analyzes the oscillating part of the dependence of the absorption coefficient on energy, extending to 400–2000 eV beyond the absorption edge. From the experimentally obtained EXAFS spectra, information about the local structure, *i.e.* coordination number *N* and bond distance *R*, is extracted by the method of nonlinear spectrum fitting for each coordination sphere around the atom under study (Bunker, 2010 ▸; Schnohr & Ridgway, 2015 ▸).

#### 3.1.1. Data acquisition on nonradioactive materials at the Mn *K*-edge

The best way to evaluate the data quality that can be recorded by a laboratory-based X-ray spectrometer is to compare it directly with synchrotron data. Figure 4 ▸ shows an Mn *K*-edge spectrum recorded for Mn_2_O_3_ by the LomonosovXAS spectrometer. The spectrum is compared with the data measured at the Kurchatov Center for Synchrotron Radiation of the National Research Center ‘Kurchatov Institute’ (Chernyshov *et al.*, 2009 ▸). The synchrotron source on the beamline is a bending magnet with the 1.7 T field of the Sibir-2 storage ring. In synchrotron radiation generation, the electron beam energy is 2.5 GeV, and the average current is 70 mA. The X-ray radiation was monochromated with a Si [111] crystal.

Measurements in the laboratory were carried out at a tube power of 10 kV at a current of 4 mA. XANES measurements were performed in the energy range 6520–6850 eV with 1 eV step size and with a constant counting time per energy point of 4 s. An Si monochromator crystal [440] was used and the Bragg angle was varied from 82.04° to 70.50° during scanning. Transmission data were acquired with sample (*I*) and without sample (*I*_0_), as well as background measurements with and without sample using the same scan parameters. The total measurement time was 5 h. For one sample, four spectra were collected and combined. The final results are shown in Fig. 4 ▸(*a*).

XANES is a unique method with a wide range of applications, including ‘fingerprint’ analysis to determine the degree of oxidation. Fingerprint analysis uses known reference samples that are measured with XANES and then their spectra are compared with unknown systems. The determination of the degree of oxidation is based on the energy required for the transition of an electron from the ground level to an empty orbital. This results in a shift of the absorption edge toward higher (oxidation) or lower (reduction) energy. The position of the edge on the XANES *K*-edge can serve as a direct indicator of the valence state. Figure 4 ▸(*b*) shows an example of reference Mn samples with different degrees of oxidation compared with metallic Mn. This demonstrates the possibility of using XANES to investigate changes in the oxidation degree. In addition to the fingerprint method, more sophisticated analytical methods are used that allow a linear combination fit of known standards to estimate the relative contributions in mixed systems. This expands the analytical capabilities and allows for a more accurate characterization of mixed systems.

The EXAFS data analysis included the standard procedures of background subtraction, normalization, transformation to *k*-space [Fig.4(*c*)] and Fourier transform to *R*-space [Fig. 4(*d*)]. A non-linear least-squares algorithm was applied to fit the EXAFS curve. We used *k*-space in the region 2–8 Å^–1^ and *R*-space in the region 1–3.2 Å. Structural parameters (coordination number *N*, bond distance *R*, Debye–Waller factor σ) were determined by the best comparison of experimental EXAFS data with theoretical calculations using the *Artemis* program of the *IFEFFIT* package (Ravel & Newville, 2005 ▸). The best fit parameters for data recorded on Mn_2_O_3_ at the Mn *K*-edge (*R*, *N* and σ) are listed in Table 3 ▸. The data and fitting results from the synchrotron and LomonosovXAS spectrometer are in perfect agreement, indicating the possibility of collecting high-quality data from a laboratory-based spectrometer.

#### 3.1.2. Data acquisition on radioactive materials at the Th, U, Pu and Np *L*_3_-edges

The variety of unique physical and chemical properties of actinide systems is due to the complexity of their 5*f* electronic structure. Any method that can show the possibility of obtaining additional information about changes in the electronic structure in various actinide systems is of great interest. The advantages of laboratory X-ray spectrometer and XAS/XES methods for actinide science are listed in the *Introduction* [Sec sec1], but it should be mentioned that such instruments can bring benefits only if they are installed in a laboratory that is licensed to handle radioactive materials. The Radiochemistry Division of LMSU has a license to handle radioactive samples. Integrated with the safety protocols installed at the division, it allows XAS/XES measurements to be conducted of concentrated solutions of actinides (0.1–100 g l^−1^) or their substantial amounts in the solid state (up to grams).

As mentioned earlier, the optical scheme of the laboratory spectrometer allows the sample to be placed in front of the detector or in front of the source. The measurement time on laboratory spectrometers is much longer than on a synchrotron as a smaller photon flux is detected, therefore one can observe the effects of radiation damage on samples; this effect is especially evident during the measuring of solutions. To reduce the effects of radiation damage during experiments, it is better to place samples (especially in solutions form) on the detector. In this case the sample will be exposed to a monochromatic beam. Below we report results and highlight the potential of laboratory spectrometers, based on examples of Th-, U-, Np- and Pu-containing materials.

**Figure 5:**
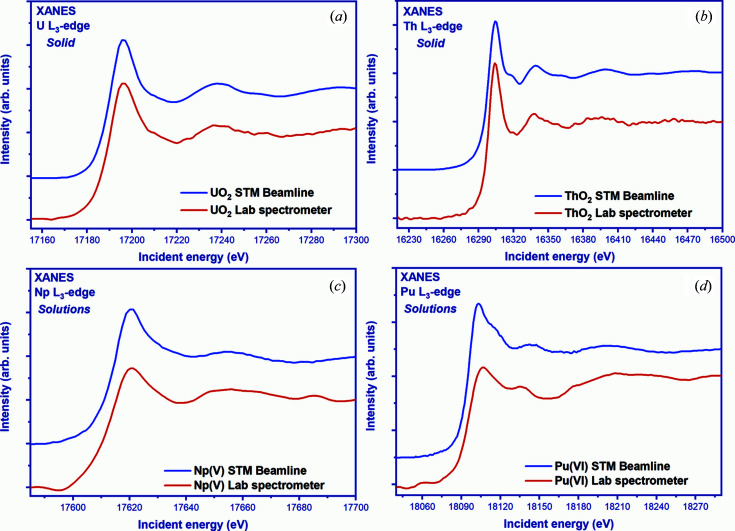
Comparison of the experimental XANES spectra of UO_2_, ThO_2_, NpO_2_^+^ and PuO_2_^2+^, recorded at the U *L*_3_-, Th *L*_3_-, Np *L*_3_- and Pu *L*_3_-edges, respectively, with help of the laboratory spectrometer (red) and at the synchrotron (blue).

The U *L*_3_ XANES on UO_2_ [Fig. 5 ▸(*a*)] was recorded in transmission mode. The X-ray beam size was limited to ∼6 mm (vertical) and ∼6 mm (horizontal) using slits mounted at the exit of the X-ray tube. The energy of the radiation was chosen using the [9 9 9] reflection of a single spherically curved Ge crystal placed at a Bragg angle of 84° at an energy of 17166 eV, which corresponds to the tabulated value of the *L*_3_ uranium absorption edge. The energy range 17150–17300 eV (Bragg angle range, in this case 84.87–80.84°) has been scanned in step sizes of 1 eV, resulting in 200 measured points with a counting time of 10 s per point. The scan time per spectrum was 33 min and the entire measurement took about 9 h. The detector count rate without a sample is 4000 photons s^−1^, so despite the small count rate the spectrum collected in the laboratory is in perfect agreement with the synchrotron data.

To record Th *L*_3_-edge XANES [Fig. 5 ▸(*b*)], the spectrometer was tuned using a silicon crystal-monochromator [10100] reflection at a Bragg angle of 82.04°. Scans were performed over an energy range of 16200 eV to 16500 eV (the Bragg angle for this changed from 83.25° to 78.05°) in 1 eV increments with a counting time of 10 s per energy point. The sample was placed in front of the detector and confined to slits along the size of the sample to reduce background noise. A total of four measurements were taken for each signal including the background measurements, which were summed to improve the signal-to-noise ratio. The procedure of multiple measurements averaging overcomes the low X-ray beam flux by increasing the time. The deviation is proportional to the square root of the number of measurements, *i.e.* four repeated measurements will improve the signal-to-noise ratio by half.

Figures 5 ▸(*c*) and 5(*d*) show the *L*_3_-edge XANES spectra of neptunium NpO_2_^+^ and plutonium PuO_2_^2+^ solutions. The samples were measured at room temperature using a plastic cuvette (10 mm × 10 mm × 1 mm) and placed in front of the detector to avoid damage to the solutions from prolonged exposure to the X-ray beam. XANES measurements were performed in transmission mode, with the X-ray beam monochromated using a Ge [1600] monochromator at a Bragg angle of 84.62° (tabulated *L*_3_-edge absorption energy 17610 eV) for Np *L*_3_ measurements, and Ge [9 9 9] for Pu *L*_3_ measurements, with a Bragg angle of 80.2° (tabulated *L*_3_-edge absorption energy 18057 eV). To obtain qualitative experimental data, ten scans were measured for each sample. A step size of 1 eV and 20 s per energy point was used to record the XANES regions. Each scan took nearly 50 min for plutonium and 36 min for neptunium, and several spectra were averaged. Table 4 ▸ gives an overview of the parameters of each XANES experiment {X-ray tube parameters, crystal-monochromator (with a radius of curvature of 0.5 m), Bragg angle, measurement time [time = (number of scans × number of points × time per point) × 4_sccans (*I*_0,_ *I*_bkg_, *I* and *I*_bkg_)]} at the U, Th, Np and Pu *L*_3_-edges.

Overall, it is shown that spectral features at the U, Th, Np and Pu *L*_3_-edges are in good agreement with synchrotron data. The higher noise level for the Th spectrum under laboratory conditions is the result of a weak sample concentration, about 6 wt%, as well as a lower flux (several orders of magnitude lower than synchrotron flux). When considering the post-edge region, a decrease is revealed in the shoulder size immediately after the white line. A possible explanation for this phenomenon is given by Amidani *et al.* (2019 ▸), relating to the size of the oxide study materials. Another explanation relates to the sample preparation (the same phenomenon was observed for Pu samples as we discuss below) due to the different sample holders and sample thicknesses. There are some discrepancies in the pre-edge region of the Pu spectrum which might be caused by the material of the sample holder contributing to the spectrum. Additional sample shielding and new sample holder should be able to correct these nonlinear effects.

### 3.2. XES configuration

In XES, a core electron absorbs a photon and is ejected to form a core-hole, which is then filled with an electron from the upper levels with X-rays. The energy of the emitted photon is the energy difference between the electronic levels (Rehr & Albers, 2000 ▸; van Bokhoven & Lamberti, 2016 ▸; Vankó *et al.*, 2006 ▸). XES gives information about the electronic structure, in particular when measuring the emission band, which determines the type of ligand, charge and spin-related information (Rovezzi & Glatzel, 2014 ▸; Holden *et al.*, 2020 ▸; Mortensen *et al.*, 2017 ▸; Huotari *et al.*, 2008 ▸; Eeckhout *et al.*, 2009 ▸; Ditter *et al.*, 2020 ▸). XES utilizes fluorescence X-rays, in the same way as known for the X-ray fluorescence (XRF) method, but both methods provide different types of information. XES can be used to study the valence state, local symmetry and electronic transitions in a material, while XRF is widely used for elemental analysis, providing information about the elemental composition and concentration of a sample. The predominantly studied emission lines by XES are the *K*-lines (*K*α and *K*β) and they are regularly measured. Emerging from filling the 1*s* orbital hole, the emission lines with the lowest energy but the brightest emission in XES are the *K*α lines, which result from the fluorescence that occurs after the 2*p* electron fills the hole in the 1*s* nucleus. Due to the 2*p* spin–orbit coupling, the *K*α line will split into two parts: the *K*α_1_ and *K*α_2_ lines (Torres & Daz-Luque, 2012 ▸). The *K*β lines are due to the fluorescence that occurs when 3*p* electrons fill the hole in the 1*s* nucleus. Due to 3*p*–3*d* exchange interactions, the main *K*β line can be split into spectral features *K*β_1,3_ (main peak) and on the low-energy side *K*β′ (shoulder). Simply put, the greater the number of unpaired electrons, the greater the splitting of features *K*β_1,3_ and *K*β′, which makes it possible to use this spectral region as a marker of the spin state (Pollock *et al.*, 2014 ▸). However, the *K*α and *K*β lines are not available for actinide measurements on a laboratory spectrometer because the energies of these lines are above 20 keV. But the emission lines of 3*d*-transition metals lie in the energy range of the spectrometer and can be measured and studied.

The feasibility of the LomonosovXAS instrument for XES detection is shown here by several measurements on U-, Th-, Pt- and Fe-containing materials. The *L*α_1_ lines of Pt, U and Th are not near the Fermi level. The lines near the Fermi level for the considered elements are the ones which have an electron transition from highest occupied orbitals, which are very weak (for example An/Ln *L*β_5_ and *K*β_5_ for transition metals). However, in this study we selected the most intense emission line as *L*α_1_ and used it in test measurements to confirm the feasibility of XES experiments using a laboratory spectrometer.

The UO_2_ and ThO_2_ powder samples were prepared using a tablet press to form the samples before the measurements. The Pt-metal and Fe-metal foils were about 5 µm thick. Experiments were performed using 30 kV and 30 mA X-ray tube parameters and chosen based on the signal-to-noise ratio. A new sample position with respect to the analyzer (in the focus of the analyzer) must be set for each element and each time during the measurements. A laser alignment was performed prior to the X-ray alignment for each sample to ensure that the crystal-monochromator and detector moved correctly along the Rowland circle and that the sample is situated in the focus position. Table 5 ▸ shows the calculated parameters for each element as measured on a laboratory spectrometer.

**Figure 6:**
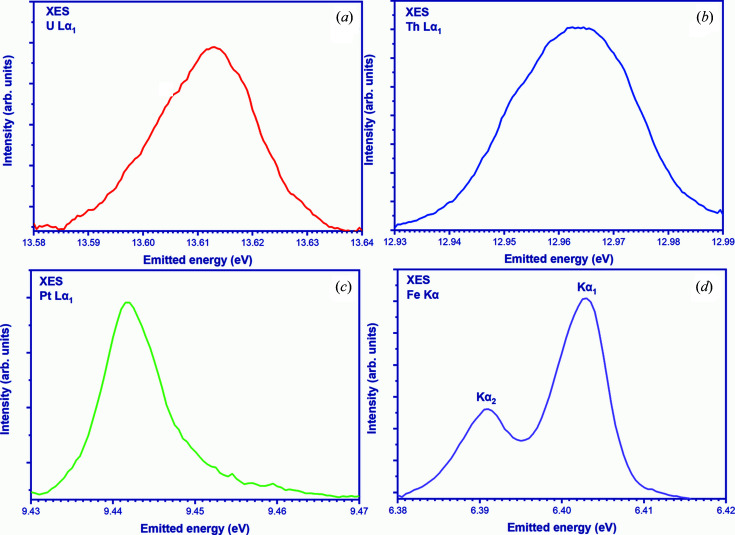
Experimental U *L*α_1_, Th *L*α_1_, Pt *L*α_1_, Fe *K*α_1_ and *K*α_2_ data recorded on (*a*) UO_2_, (*b*) ThO_2_, (*c*) Pt-metal and (*d*) Fe-metal, respectively.

Figures 6 ▸(*a*) and 6(*b*) show the *L*α_1_ emission line (13614 eV) of UO_2_ and the *L*α_1_ emission line (12968 eV) of ThO_2_, which corresponds to *L*_3_–*M*_5_ electron transitions. The emitted energy was chosen from the reflections of Ge[7 7 7] (Bragg angle of 77.39°) for U, and Ge[8 8 0] (Bragg angle of 72.9°) for Th measurements. The samples were oriented at a 45° angle with respect to the incident X-ray beam. The emitted energy was scanned in the 60 eV range with a step size of 0.5 eV and a counting time of 5 s per point for the entire energy range. Three spectra were measured for each sample and the measurement time was approximately 15 min per spectrum.

Figure 6 ▸(*c*) shows the *L*α_1_ XES recorded on the Pt foil. The XES spectrum was obtained near the emitted energy of 9442 eV using the reflection of a Ge[6 6 0] crystal oriented at a Bragg angle of 79.98° with a counting time of 3 s per point (step size 0.5 eV). Four spectra were collected and averaged. Figure 6 ▸(*d*) shows the Fe metal *K*α_1_ (*K*-*L*_3_) and *K*α_2_ (*K*-*L*_2_) XES, recorded with the help of a Ge[4 4 4] analyzer for both emission line measurements. The Bragg angle was varied from 75.86° to 75.41°, in the energy interval 6380–6420 eV (40 eV) with 0.5 eV step size. Because of the relatively low energy, a helium chamber was used to reduce fluorescence absorption. In total, four spectra of 2.5 min each were collected, resulting in a total counting time of 10 min.

Such test measurements revealed no shifts between different scans (it should be noted again that we use motorized platforms from xHuber, which have the best moving accuracy and minimum pitch error). The FWHM of XES was estimated to be 7 eV. Moreover, if we install special X-ray slits directly at the exit of the X-ray tube, this represents a potential improvement in the energy resolution during the experiments. These slits offer the possibility of fine-tuning the resolution by changing their geometric parameters, width and height. It is worth noting, however, that this entails some reduction in count rate, and an optimal compromise must be found in each individual case. Typically, *K*α_1_ allows observation of tiny energy shifts of the measured emission lines depending on the degree of oxidation of the element in the sample. This spin–orbit interaction is strong for the *K*α lines and much weaker for the *K*β lines (Lafuerza *et al.*, 2020 ▸).

## 4. Conclusions

Here we demonstrate the concept of an X-ray spectrometer in Rowland geometry based on an X-ray tube, a Johann-type crystal-monochromator and an X-ray detector. We demonstrate that it is possible to obtain data with the same energy resolution as the synchrotron in the 4–20 keV range. Moreover, we report the experimental XAS and XES data, obtained in the laboratory on actinide-containing compounds. All these experiments show that the power of the laboratory X-ray source is sufficient for both XAS and XES measurements.

Measurements have been carried out *ex situ* and all parameters, including the collection time and actinide concentrations, are shown. This laboratory X-ray spectrometer has great potential and studies reported here can expand to the number of wide actinide applications. The ability to study radioactive materials in the laboratory, immediately after the synthesis, and overcome issues related to transportation of highly active materials to synchrotrons is very appealing. It can also be used for interim studies to select the best samples for higher energy resolution synchrotron experiments. The main advantage of such instruments in the laboratory is that they are very compact and adaptable to different working conditions. This is especially important for radioactive short-lived materials, which can be synthesized and investigated immediately by XAS and XES. *In situ* reactions with actinide-containing materials, performed with laboratory spectrometers, have a great capacity. Additionally, laboratory spectrometers are an excellent tool for the professional training of students, as well as for expanding and deepening the knowledge and skills of researchers needed to expertly set up and conduct research using synchrotron sources.

The main problem with laboratory equipment is the relatively low signal-to-noise ratio, since the photon flux of the X-ray tube is several orders of magnitude smaller than that of the synchrotron, which must be compensated by long measurement times. This, to some extent, limits the study of samples with very low concentration and optically thin films, but work in this direction is ongoing and there are already publications about experiments on laboratory spectrometers in fluorescence mode and slow *in situ* reactions (Kallio *et al.*, 2022 ▸). As shown by Kallio and co-workers, laboratory spectrometers can be useful for studying slow *in situ* reactions over hours, days and weeks (Genz *et al.*, 2022 ▸, 2023 ▸).

The development and evolution of more powerful X-ray sources as well as highly efficient detectors for hard X-ray energies may lead to further developments of laboratory spectrometers and minimization of the measurement times. In addition, the size of the X-ray beam has a significant impact on the resolution of the spectrometer and, consequently, reduces the spectral resolution. The footprint of the beam on the sample leads to a finite scattering volume related to the size of the footprint. The energy resolution is given via the differential Bragg law: Δ*E* = *E*cotΘ_B_ΔΘ, with *E* being the incident energy, Θ_B_ the Bragg angle for the studied reflection and Θ the angular width of the source as seen from a point on the analysis (Collart *et al.*, 2005 ▸). This can be solved by using a microfocus X-ray source that provides an X-ray beam focused to a few tens of micrometres, sufficient for high-resolution XAS and XES experiments at the cost of reduced count rate and intensity level. Such a setup with sealed microfocus ultrahigh-brightness source with a spot size of 30–100 µm is possible to purchase from Sigray.

Overall, we show here the potential use of the laboratory X-ray spectrometers for actinide science. It is clear that significant effort has been made to perform XAS and XES experiments on actinide systems at synchrotrons, and to understand the observed phenomena. Considerable progress in understanding the geochemistry, physics, chemistry and environmentally related problems has resulted from synchrotron studies. However, the full electronic structure of actinide systems is far from complete, both from experimental and theoretical viewpoints; many possibilities exist for active research for many years to come. We believe that more progress will be made with the help of laboratory spectrometers, installed in licensed radiochemistry divisions worldwide.

## Figures and Tables

**Table 1:** List of principal parameters of the spectrometer with a 0.5 m Rowland cycle

Parameter	Range
Photon energy (keV)	4–20
Bragg angle Θ (°)	65–85
Distance from monochromator crystal to curvature radius source, 0.5 m (mm)	450–500
detx drive range (mm)	0–400
dety drive range (mm)	0–200
detrot rotation range 2Θ (mm)	130–172

**Table 2:** Activity of the investigated samples

Sample	Radioactivity (Bq)
UO_2_	110
ThO_2_	80
NpO_2_^+^	49.5 × 10^3^
PuO_2_^2+^	4 × 10^6^

**Table 3:** Metric parameters extracted by least-squares fit analysis of Mn *K*-edge EXAFS spectra (*k*-range)

	*R* (Å)		σ (Å^2^)		
Path	Laboratory	STM	Laboratory	STM	*N*
Mn–O	1.91	1.90	0.003	0.002	4
Mn–O	2.16	2.15	0.002	0.002	2
Mn–Mn	3.09	3.07	0.007	0.007	6
Mn–Mn	3.66	3.61	0.012	0.012	6

**Table 4:** Overview of the parameters of the XAS experiment at the U, Th, Np and Pu *L*_3_-edges

Sample	Energy (eV)	X-ray tube parameters	Crystal (0.5 m)	Θ *L*_3_-edge	Edge steps	Number of scans (*I*)	Seconds per energy point	Amount of points	Time
ThO_2_	16283	28 kV, 10 mA	Si 10 10 0	82.47°	0.5	4	10	300	13 h
UO_2_	17166	30 kV, 10 mA	Ge 9 9 9	84.31°	0.96	4	10	200	9 h
NpO_2_^+^	17610	35 kV, 10 mA	Ge 16 0 0	84.62°	0.43	10	20	220	48 h
PuO_2_^2+^	18057	35 kV, 10 mA	Si 9 9 9	80.2°	1.06	10	20	300	66 h

**Table 5:** Estimated parameters for Th *L*α_1_, U *L*α_1_, F *K*α_1,_ *K*α_2_ and Pt *L*α_1_ XES data collection with the LomonosovXAS spectrometer

Number	Sample	Energy (eV)	Emission line	Crystal monochromator (0.5 m)	Bragg angle	Distance from crystal to detector (mm)
1	ThO_2_	12968	*L*α_1_	Ge 880	72.93°	477.997
2	UO_2_	13614	*L*α_1_	Ge 777	77.39°	487.399
3	Fe-metal	6405	*K*α_1_	Ge 440	75.41°	483.877
		6392	*K*α_2_	Ge 440	75.86°	484.869
5	Pt-metal	9442	*L*α_1_	Ge 660	79.98°	492.374
